# Supplementary material for: Structural-functional multilayer brain network properties and outcome of combined repetitive transcranial magnetic stimulation and psychotherapy for obsessive–compulsive disorder
Source: Neuroimage Clin. 2026 May 26;50:104012. doi: 10.1016/j.nicl.2026.104012 (PMC13241641; doi:10.1016/j.nicl.2026.104012)
Supplement: Supplementary Data 1 [file mmc1.pdf]

Fitzsimmons et al. Structural-functional multilayer brain network properties and outcome of combined repetitive transcranial magnetic stimulation and psychotherapy for obsessive-compulsive disorder

Supplementary information

Supplementary Methods

1. *Interventions:*

rTMS/ERP intervention and blinding

Randomization was stratified based on current use of SRI. Randomization was carried out following baseline assessments by a researcher not involved in clinical assessments. After randomization patients received either: 10Hz rTMS at 110% resting motor threshold (RMT) to the left DLPFC; 10Hz rTMS at 110% RMT to the left preSMA; or 10Hz rTMS at 60% RMT to the vertex. All participants received 3000 pulses in 30x10s trains with 30 seconds intertrain intervals[1].

The resting motor threshold (RMT) of the left motor cortex was determined once, within 2 weeks prior to starting rTMS treatment. Single TMS pulses were applied, gradually increasing in intensity, until five out of 10 pulses resulted in a right index finger motor-evoked potential of at least 50μV as measured using electromyography of the first dorsal interosseus muscle.

ERP sessions started within 10 minutes after the rTMS session was completed; each session lasted 60 minutes. Psychotherapists followed a protocolized treatment manual, including two introductory sessions, 16 exposure sessions (twice per week, directly following rTMS), one evaluation session, and one relapse prevention session, within ~10 weeks. Each patient worked with two different therapists during the treatment, and therapists received monthly supervision by an expert in CBT for OCD. Exposure sessions consisted of therapist-aided exposure exercises, and self-guided exposure exercises were assigned as between-session homework.

Participants, psychotherapists and clinical assessors were blinded to rTMS condition; rTMS therapists were unblinded but were instructed not to discuss the treatment conditions with participants or psychotherapists and did not take part in clinical assessments or ERP sessions.

Creation of stimulation targets

Please see Fitzsimmons et al[2] for full details of fMRI acquisition and processing for rTMS target creation, and for a full description of the Tower of London and Stop-Signal task cognitive tasks. For the DLPFC condition, the peak voxel in the left DLPFC (defined as Brodmann areas 9 and 46[3]) during the planning contrast of the Tower of London task (all planning conditions > baseline) was determined, and the stimulation target placed on the closest gyrus in the participant's T1 anatomical scan. For the preSMA condition, the local maximum of activation in the left preSMA (defined as the medial gyrus of Brodmann area 6 anterior to the anterior commissure[4] during the response inhibition contrast (successful stop trials > successful go trials) of the stop-signal task were used to determine the stimulation target for the preSMA condition. If no suitable local maximum could be located for the DLPFC or preSMA groups at a statistical threshold of P<0.01 uncorrected, the following literature coordinates (in MNI space) were used: i. Left DLPFC: -40, 28, 30[5]ii. Left preSMA: -4, 14, 58[6] For all participants in the vertex group the following MNI coordinates were used: 0, -34, 72[1] Once the individualized stimulation coordinate was defined, a 5mm ROI was created, warped from MNI to subject space and overlaid on a T1 MRI scan of the individual participant, allowing navigation to the individualized stimulation location at every rTMS treatment session using the Localite neuronavigation system (Localite GmbH, Bonn, Germany)

2. Detailed fMRIprep preprocessing boilerplate for rsfMRI data

Results included in this manuscript come from preprocessing performed using fMRIPrep 21.0.1[7,8]; RRID:SCR_016216, which is based on Nipype 1.6.1[9,10]; RRID:SCR_002502.

Preprocessing of B0 inhomogeneity mappings

A total of 2 fieldmaps were available per subject. A B0-nonuniformity map (or fieldmap) was estimated based on two (or more) echo-planar imaging (EPI) references with topup ([11]; FSL 6.0.5.1:57b01774).

Anatomical data preprocessing

A total of 2 T1-weighted (T1w) images were found within the input BIDS dataset. All of them were corrected for intensity non-uniformity (INU) with N4BiasFieldCorrection [12], distributed with ANTs 2.3.3 ([13], RRID:SCR_004757). The T1w-reference was then skull-stripped with a Nipype implementation of the antsBrainExtraction.sh workflow (from ANTs), using OASIS30ANTs as target template. Brain tissue segmentation of cerebrospinal fluid (CSF), white-matter (WM) and gray-matter (GM) was performed on the brain-extracted T1w using fast (FSL 6.0.5.1:57b01774, RRID:SCR_002823, [14]). A T1w-reference map was computed after registration of 2 T1w images (after INU-correction) using mri_robust_template (FreeSurfer 6.0.1, [15]). Brain surfaces were reconstructed using recon-all (FreeSurfer 6.0.1, RRID:SCR_001847,[16]), and the brain mask estimated previously was refined with a custom variation of the method to reconcile ANTs-derived and FreeSurfer-derived segmentations of the cortical gray-matter of Mindboggle (RRID:SCR_002438,[17]). Volume-based spatial normalization to two standard spaces (MNI152NLin6Asym, MNI152NLin2009cAsym) was performed through nonlinear registration with antsRegistration (ANTs 2.3.3), using brain-extracted versions of both T1w reference and the T1w template. The following templates were selected for spatial normalization: FSL's MNI ICBM 152 non-linear 6th Generation Asymmetric Average Brain Stereotaxic Registration Model [[18], RRID:SCR_002823; TemplateFlow ID: MNI152NLin6Asym], ICBM 152 Nonlinear Asymmetrical template version 2009c [[19], RRID:SCR_008796; TemplateFlow ID: MNI152NLin2009cAsym].

Functional data preprocessing

For each of the 4 BOLD runs per subject (across all tasks and sessions), the following preprocessing was performed. First, a reference volume and its skull-stripped version were generated using a custom methodology of fMRIPrep. Head-motion parameters with respect to the BOLD reference (transformation matrices, and six corresponding rotation and translation parameters) are estimated before any spatiotemporal filtering using mcflirt (FSL 6.0.5.1:57b01774,[20]). The estimated fieldmap was then aligned with rigid-registration to the target EPI (echo-planar imaging) reference run. The field coefficients were mapped on to the

reference EPI using the transform. BOLD runs were slice-time corrected to 1.07s (0.5 of slice acquisition range 0s-2.15s) using 3dTshift from AFNI ([21], RRID:SCR_005927). The BOLD reference was then co-registered to the T1w reference using bbregister (FreeSurfer) which implements boundary-based registration[22]. Co-registration was configured with six degrees of freedom. Several confounding time-series were calculated based on the preprocessed BOLD: framewise displacement (FD), DVARS and three region-wise global signals. FD was computed using two formulations following Power (absolute sum of relative motions,[23]) and Jenkinson (relative root mean square displacement between affines,[20]). FD and DVARS are calculated for each functional run, both using their implementations in Nipype (following the definitions by [23]). The three global signals are extracted within the CSF, the WM, and the whole-brain masks. Additionally, a set of physiological regressors were extracted to allow for component-based noise correction (CompCor,[24]). Principal components are estimated after high-pass filtering the preprocessed BOLD time-series (using a discrete cosine filter with 128s cut-off) for the two CompCor variants: temporal (tCompCor) and anatomical (aCompCor). tCompCor components are then calculated from the top 2% variable voxels within the brain mask. For aCompCor, three probabilistic masks (CSF, WM and combined CSF+WM) are generated in anatomical space. The implementation differs from that of Behzadi et al. in that instead of eroding the masks by 2 pixels on BOLD space, the aCompCor masks are subtracted a mask of pixels that likely contain a volume fraction of GM. This mask is obtained by dilating a GM mask extracted from the FreeSurfer's aseg segmentation, and it ensures components are not extracted from voxels containing a minimal fraction of GM. Finally, these masks are resampled into BOLD space and binarized by thresholding at 0.99 (as in the original implementation). Components are also calculated separately within the WM and CSF masks. For each CompCor decomposition, the k components with the largest singular values are retained, such that the retained components' time series are sufficient to explain 50 percent of variance across the nuisance mask (CSF, WM, combined, or temporal). The remaining components are dropped from consideration. The head-motion estimates calculated in the correction step were also placed within the corresponding confounds file. The confound time series derived from head motion estimates and global signals were expanded with the inclusion of temporal derivatives and quadratic terms for each [25]. Frames that exceeded a threshold of 0.5

mm FD or 1.5 standardised DVARS were annotated as motion outliers. The BOLD time-series were resampled into standard space, generating a preprocessed BOLD run in MNI152NLin6Asym space. First, a reference volume and its skull-stripped version were generated using a custom methodology of fMRIPrep. The BOLD time-series were resampled onto the following surfaces (FreeSurfer reconstruction nomenclature): fsnative, fsaverage5. Automatic removal of motion artifacts using independent component analysis (ICA-AROMA, [26]) was performed on the preprocessed BOLD on MNI space time-series after removal of non-steady state volumes and spatial smoothing with an isotropic, Gaussian kernel of 6mm FWHM (full-width half-maximum). Corresponding “non-aggresively” denoised runs were produced after such smoothing. Additionally, the “aggressive” noise-regressors were collected and placed in the corresponding confounds file. All resamplings can be performed with a single interpolation step by composing all the pertinent transformations (i.e. head-motion transform matrices, susceptibility distortion correction when available, and co-registrations to anatomical and output spaces). Gridded (volumetric) resamplings were performed using antsApplyTransforms (ANTs), configured with Lanczos interpolation to minimize the smoothing effects of other kernels [27]. Non-gridded (surface) resamplings were performed using mri_vol2surf (FreeSurfer).

Many internal operations of fMRIPrep use Nilearn 0.8.1 ([28], RRID:SCR_001362), mostly within the functional processing workflow. For more details of the pipeline, see the section corresponding to workflows in fMRIPrep’s documentation.

Copyright Waiver

The above boilerplate text was automatically generated by fMRIPrep with the express intention that users should copy and paste this text into their manuscripts unchanged. It is released under the CC0 license.

3. Excluded Schaefer 300 atlas[29] regions

7Networks_LH_Limbic_OFC_3

7Networks_LH_Limbic_TempPole_1

7Networks_LH_Limbic_TempPole_4

7Networks_LH_Cont_OFC_1

7Networks_RH_Limbic_OFC_3

7Networks_RH_Limbic_TempPole_1

7Networks_RH_Limbic_TempPole_4

References

[1] de Wit SJ, van der Werf YD, Mataix-Cols D, Trujillo JP, van Oppen P, Veltman DJ, et al. Emotion regulation before and after transcranial magnetic stimulation in obsessive compulsive disorder. Psychol Med 2015;45:3059–73. https://doi.org/10.1017/S0033291715001026.

[2] Fitzsimmons SMDD, Postma T, van Campen AD, Vriend C, Batelaan NM, van Oppen P, et al. TMS-induced plasticity improving cognitive control in OCD I: Clinical and neuroimaging outcomes from a randomised trial of rTMS for OCD. medRxiv 2023:2023.11.04.23298100. https://doi.org/10.1101/2023.11.04.23298100.

[3] Sanches M, Caetano S, Nicoletti M, Monkul ES, Chen HH, Hatch JP, et al. An MRI-based approach for the measurement of the dorsolateral prefrontal cortex in humans. Psychiatry Res 2009;173:150–4. https://doi.org/10.1016/j.pscychresns.2009.02.007.

[4] Picard N, Strick PL. Imaging the premotor areas. Curr Opin Neurobiol 2001;11:663–72. https://doi.org/10.1016/s0959-4388(01)00266-5.

[5] Trujillo JP, Gerrits NJHM, Vriend C, Berendse HW, van den Heuvel OA, van der Werf YD. Impaired planning in Parkinson’s disease is reflected by reduced brain activation and connectivity. Human Brain Mapping 2015;36:3703–15. https://doi.org/10.1002/hbm.22873.

[6] Norman LJ, Taylor SF, Liu Y, Radua J, Chye Y, De Wit SJ, et al. Error Processing and Inhibitory Control in Obsessive-Compulsive Disorder: A Meta-analysis Using Statistical Parametric Maps. Biological Psychiatry 2019;85:713–25. https://doi.org/10.1016/j.biopsych.2018.11.010.

[7] Esteban O, Markiewicz CJ, Blair RW, Moodie CA, Isik AI, Erramuzpe A, et al. fMRIPrep: a robust preprocessing pipeline for functional MRI. Nat Methods 2019;16:111–6. https://doi.org/10.1038/s41592-018-0235-4.

[8] Esteban O, Markiewicz CJ, Goncalves M, Provins C, Kent JD, DuPre E, et al. fMRIPrep: a robust preprocessing pipeline for functional MRI 2023. https://doi.org/10.5281/ZENODO.852659.

[9] Gorgolewski K, Burns CD, Madison C, Clark D, Halchenko YO, Waskom ML, et al. Nipype: A Flexible, Lightweight and Extensible Neuroimaging Data Processing Framework in Python. Front Neuroinform 2011;5. https://doi.org/10.3389/fninf.2011.00013.

[10] Esteban O, Markiewicz CJ, Burns C, Goncalves M, Jarecka D, Ziegler E, et al. nipy/nipype: 1.8.3 2022. https://doi.org/10.5281/ZENODO.596855.

[11] Andersson JLR, Skare S, Ashburner J. How to correct susceptibility distortions in spin-echo echo-planar images: application to diffusion tensor imaging. NeuroImage 2003;20:870–88. https://doi.org/10.1016/S1053-8119(03)00336-7.

[12] Tustison NJ, Avants BB, Cook PA, Yuanjie Zheng, Egan A, Yushkevich PA, et al. N4ITK:

Improved N3 Bias Correction. IEEE Trans Med Imaging 2010;29:1310–20. https://doi.org/10.1109/TMI.2010.2046908.
[13] Avants B, Epstein C, Grossman M, Gee J. Symmetric diffeomorphic image registration with cross-correlation: Evaluating automated labeling of elderly and neurodegenerative brain. Medical Image Analysis 2008;12:26–41. https://doi.org/10.1016/j.media.2007.06.004.
[14] Zhang Y, Brady M, Smith S. Segmentation of brain MR images through a hidden Markov random field model and the expectation-maximization algorithm. IEEE Trans Med Imaging 2001;20:45–57. https://doi.org/10.1109/42.906424.
[15] Reuter M, Rosas HD, Fischl B. Highly accurate inverse consistent registration: A robust approach. NeuroImage 2010;53:1181–96. https://doi.org/10.1016/j.neuroimage.2010.07.020.
[16] Dale AM, Fischl B, Sereno MI. Cortical Surface-Based Analysis. NeuroImage 1999;9:179–94. https://doi.org/10.1006/nimg.1998.0395.
[17] Klein A, Ghosh SS, Bao FS, Giard J, Häme Y, Stavsky E, et al. Mindboggling morphometry of human brains. PLoS Comput Biol 2017;13:e1005350. https://doi.org/10.1371/journal.pcbi.1005350.
[18] Evans AC, Janke AL, Collins DL, Baillet S. Brain templates and atlases. NeuroImage 2012;62:911–22. https://doi.org/10.1016/j.neuroimage.2012.01.024.
[19] Fonov V, Evans A, McKinstry R, Almli C, Collins D. Unbiased nonlinear average age-appropriate brain templates from birth to adulthood. NeuroImage 2009;47:S102. https://doi.org/10.1016/S1053-8119(09)70884-5.
[20] Jenkinson M, Bannister P, Brady M, Smith S. Improved optimization for the robust and accurate linear registration and motion correction of brain images. Neuroimage 2002;17:825–41.
[21] Cox RW, Hyde JS. Software tools for analysis and visualization of fMRI data. NMR Biomed 1997;10:171–8. https://doi.org/10.1002/(SICI)1099-1492(199706/08)10:4/5<171::AID-NBM453>3.0.CO;2-L.
[22] Greve DN, Fischl B. Accurate and robust brain image alignment using boundary-based registration. NeuroImage 2009;48:63–72. https://doi.org/10.1016/j.neuroimage.2009.06.060.
[23] Power JD, Mitra A, Laumann TO, Snyder AZ, Schlaggar BL, Petersen SE. Methods to detect, characterize, and remove motion artifact in resting state fMRI. NeuroImage 2014;84:320–41. https://doi.org/10.1016/j.neuroimage.2013.08.048.
[24] Behzadi Y, Restom K, Liau J, Liu TT. A component based noise correction method (CompCor) for BOLD and perfusion based fMRI. NeuroImage 2007;37:90–101. https://doi.org/10.1016/j.neuroimage.2007.04.042.
[25] Satterthwaite TD, Elliott MA, Gerraty RT, Ruparel K, Loughead J, Calkins ME, et al. An improved framework for confound regression and filtering for control of motion artifact in the preprocessing of resting-state functional connectivity data. NeuroImage 2013;64:240–56. https://doi.org/10.1016/j.neuroimage.2012.08.052.
[26] Pruim RHR, Mennes M, Van Rooij D, Llera A, Buitelaar JK, Beckmann CF. ICA-AROMA: A robust ICA-based strategy for removing motion artifacts from fMRI data. NeuroImage 2015;112:267–77. https://doi.org/10.1016/j.neuroimage.2015.02.064.
[27] Lanczos C. Evaluation of Noisy Data. Journal of the Society for Industrial and Applied Mathematics Series B Numerical Analysis 1964;1:76–85. https://doi.org/10.1137/0701007.
[28] Abraham A, Pedregosa F, Eickenberg M, Gervais P, Mueller A, Kossaifi J, et al. Machine learning for neuroimaging with scikit-learn. Front Neuroinform 2014;8. https://doi.org/10.3389/fninf.2014.00014.
[29] Schaefer A, Kong R, Gordon EM, Laumann TO, Zuo X-N, Holmes AJ, et al. Local-Global Parcellation of the Human Cerebral Cortex from Intrinsic Functional Connectivity MRI. Cereb Cortex 2018;28:3095–114. https://doi.org/10.1093/cercor/bhx179.

Supplementary tables

Table S1: stimulated resting state networks per treatment group

	**DLPFC (N)**	**preSMA (N)**	**vertex-DLPFC (N)**	**vertex-preSMA (N)**
**Frontoparietal**	**14**	**4**	**13**	**2**
**Default Mode**	**5**	**16**	**5**	**7**
**Ventral Attention**	**0**	**0**	**0**	**9**
**Somatomotor**	**0**	**1**	**0**	**0**

Table S2: results of multiple regressions predicting clinical outcome from graph properties of stimulation locations derived from single-layer structural networks. Coefficients for T0-T2 delta YBOCS, adjusted for age, sex and T0 YBOCS score, are shown.

group	Predictor	Coefficient	Standard Error	P value	Adjusted R2 for model	P value of model	group	Predictor	Coefficient	Standard Error	P value	Adjusted R2 for model	P value of model
DLPFC	BC of stimulated node	0.001	0.001	0.255	-0.064	0.586	Vertex-DLPFC	BC of stimulated node	0.001	0.001	0.441	0.033	0.378
	PC of stimulated node	0.007	0.005	0.206	-0.097	0.667		PC of stimulated node	-0.004	0.008	0.589	0.129	0.226
	ECof stimulated node	0.000	0.001	0.882	-0.261	0.990		ECof stimulated node	0.001	0.001	0.275	0.348	0.046
	NS of stimulated node	0.000	0.000	0.365	-0.052	0.557		NS of stimulated node	0.000	0.000	0.375	0.047	0.354
	Mean BC of stimulated network	0.000	0.000	0.876	-0.062	0.581		Mean BC of stimulated network	0.000	0.000	0.168	-0.091	0.639
	Mean PC of stimulated network	0.000	0.000	0.225	0.103	0.251		Mean PC of stimulated network	0.000	0.000	0.076	0.000	0.442
	Mean EC of stimulated network	0.000	0.002	0.900	-0.131	0.751		Mean EC of stimulated network	0.004	0.002	0.056	0.220	0.126
	Mean NS of stimulated network	0.000	0.000	0.470	-0.068	0.595		**Mean NS of stimulated network**	**0.000**	**0.000**	**0.036***	**0.093**	**0.278**
preSMA	BC of stimulated node	0.000	0.001	0.861	0.197	0.112	Vertex-preSMA	BC of stimulated node	-0.001	0.002	0.535	-0.051	0.549
	PC of stimulated node	-0.003	0.003	0.322	-0.103	0.713		PC of stimulated node	0.002	0.004	0.564	0.139	0.213
	ECof stimulated node	0.000	0.001	0.896	-0.025	0.499		ECof stimulated node	0.000	0.001	0.720	0.161	0.186
	NS of stimulated node	0.000	0.000	0.850	-0.104	0.717		NS of stimulated node	-0.001	0.000	0.124	0.223	0.124
	Mean BC of stimulated network	0.000	0.000	0.664	-0.035	0.526		Mean BC of stimulated network	0.000	0.000	0.471	0.006	0.431
	Mean PC of stimulated network	0.000	0.000	0.821	-0.102	0.710		Mean PC of stimulated network	0.000	0.000	0.338	-0.084	0.625
	Mean EC of stimulated network	-0.001	0.002	0.732	-0.038	0.532		Mean EC of stimulated network	0.001	0.002	0.607	-0.148	0.770
	Mean NS of stimulated network	0.000	0.000	0.791	-0.234	0.994		**Mean NS of stimulated network**	**0.000**	**0.000**	**0.049***	**0.194**	**0.150**

BC, betweenness centrality; PC, participation coefficient; EC, eigenvector centrality; NS, node strength; DLPFC, dorsolateral prefrontal cortex; preSMA, pre-supplementary motor area; *p<0.05 but does not survive correction for multiple comparisons with α=0.019

Table S3: results of multiple regressions predicting clinical outcome from graph properties of stimulation locations derived from single-layer functional networks. Coefficients for T0-T2 delta YBOCS, adjusted for age, sex and T0 YBOCS score, are shown.

group	Predictor	Coefficient	Standard Error	P value	Adjusted R2 for model	P value of model	group	Predictor	Coefficient	Standard Error	P value	Adjusted R2 for model	P value of model
DLPFC	BC of stimulated node	0.000	0.000	0.458	0.210	0.123	Vertex-DLPFC	BC of stimulated node	0.000	0.000	0.160	-0.001	0.444
	PC of stimulated node	-0.001	0.002	0.736	-0.107	0.692		PC of stimulated node	0.004	0.003	0.256	-0.066	0.583
	ECof stimulated node	0.000	0.000	0.477	0.062	0.318		EC of stimulated node	0.001	0.001	0.086	0.260	0.095
	NS of stimulated node	0.001	0.002	0.498	-0.180	0.864		NS of stimulated node	0.003	0.002	0.155	0.158	0.189
	Mean BC of stimulated network	0.000	0.000	0.634	0.085	0.279		Mean BC of stimulated network	0.000	0.000	0.500	0.014	0.415
	Mean PC of stimulated network	0.000	0.000	0.525	0.058	0.325		Mean PC of stimulated network	0.000	0.000	0.265	0.000	0.443
	Mean EC of stimulated network	-0.001	0.002	0.559	-0.206	0.916		Mean EC of stimulated network	0.002	0.001	0.152	0.165	0.181
	Mean NS of stimulated network	0.001	0.001	0.421	-0.206	0.916		Mean NS of stimulated network	0.000	0.001	0.792	-0.209	0.895
preSMA	BC of stimulated node	0.000	0.000	0.417	-0.056	0.582	Vertex-preSMA	BC of stimulated node	0.000	0.000	0.821	0.066	0.320
	PC of stimulated node	-0.002	0.002	0.515	-0.145	0.830		PC of stimulated node	0.003	0.003	0.239	-0.126	0.720
	EC of stimulated node	0.000	0.000	0.720	-0.035	0.525		ECof stimulated node	0.001	0.001	0.271	-0.064	0.579
	NS of stimulated node	0.001	0.002	0.362	0.066	0.294		NS of stimulated node	0.002	0.003	0.547	-0.043	0.531
	Mean BC of stimulated network	0.000	0.000	0.601	-0.103	0.714		Mean BC of stimulated network	0.000	0.000	0.083	0.271	0.087
	Mean PC of stimulated network	0.000	0.000	0.333	-0.080	0.649		Mean PC of stimulated network	0.000	0.000	0.336	-0.037	0.519
	Mean EC of stimulated network	-0.001	0.001	0.525	-0.101	0.707		Mean EC of stimulated network	0.001	0.002	0.637	0.015	0.414
	Mean NS of stimulated network	0.001	0.001	0.554	-0.182	0.918		Mean NS of stimulated network	0.001	0.003	0.846	-0.150	0.773

BC, betweenness centrality; PC, participation coefficient; EC, eigenvector centrality; NS, node strength; DLPFC, dorsolateral prefrontal cortex; preSMA, pre-supplementary motor area

Table S4: results of multiple regressions predicting clinical outcome from graph properties of stimulation locations derived from multilayer functional-structural networks. Coefficients for T0-T2 delta YBOCS, adjusted for age, sex and T0 YBOCS score, are shown.

group	Predictor	Coefficient	Standard Error	P value	Adjusted R2 for model	P value of model	group	Predictor	Coefficient	Standard Error	P value	Adjusted R2 for model	P value of model
DLPFC	BC of stimulated node	0.005	0.004	0.199	-0.108	0.693	Vertex-DLPFC	BC of stimulated node	0.003	0.008	0.708	-0.134	0.738
	EC of stimulated node	0.004	0.005	0.417	-0.131	0.752		EC of stimulated node	-0.001	0.002	0.583	-0.158	0.791
	DC of stimulated node	0.005	0.004	0.175	-0.004	0.449		DC of stimulated node	0.003	0.008	0.734	-0.062	0.574
	**Mean BC of stimulated network**	**0.004**	**0.002**	**0.013****	**0.391**	**0.025**		Mean BC of stimulated network	-0.004	0.002	0.143	0.176	0.169
	Mean EC of stimulated network	0.002	0.002	0.225	-0.116	0.714		Mean EC of stimulated network	-0.004	0.002	0.053	0.109	0.253
	Mean DC of stimulated network	0.002	0.002	0.325	0.035	0.368		Mean DC of stimulated network	-0.003	0.003	0.291	0.046	0.356
preSMA	BC of stimulated node	-0.004	0.004	0.335	-0.122	0.767	Vertex-preSMA	BC of stimulated node	0.000	0.006	0.948	-0.140	0.752
	EC of stimulated node	-0.003	0.004	0.500	0.002	0.432		EC of stimulated node	0.002	0.005	0.741	0.013	0.416
	DC of stimulated node	0.003	0.003	0.364	-0.053	0.574		DC of stimulated node	0.002	0.005	0.679	0.311	0.063
	Mean BC of stimulated network	-0.001	0.001	0.639	-0.134	0.800		Mean BC of stimulated network	-0.001	0.002	0.562	0.068	0.318
	Mean EC of stimulated network	0.002	0.002	0.290	-0.158	0.861		Mean EC of stimulated network	-0.003	0.002	0.117	0.026	0.392
	Mean DC of stimulated network	0.002	0.003	0.445	-0.066	0.609		Mean DC of stimulated network	-0.001	0.003	0.597	0.001	0.441

BC, betweenness centrality; EC, eigenvector centrality; DC, degree centrality; DLPFC, dorsolateral prefrontal cortex; preSMA, pre-supplementary motor area; **=survives correction for multiple comparisons with p<0.025

Table S5: results of post hoc multiple regressions predicting clinical outcome from graph properties of stimulation locations derived from multilayer functional-structural networks in the DLPFC group. Coefficients for T0-T2 delta YBOCS, adjusted for age, sex and T0 YBOCS score, are shown.

group	Predictor	Coefficient	Standard Error	P value	Adjusted R2 for model	P value of model
DLPFC	Wholebrain mean BC	0.003	0.001	0.076	0.196	0.136
	Wholebrain mean BC without DMN and FPN	0.002	0.001	0.175	0.101	0.254
	Mean BC of somatomotor network	0.000	0.001	0.747	0.002	0.436

BC, betweenness centrality; DMN, default mode network; FPN, frontoparietal network
